# Statistical Analyses of Femur Parameters for Designing Anatomical Plates

**DOI:** 10.1155/2016/1247560

**Published:** 2016-12-01

**Authors:** Lin Wang, Kunjin He, Zhengming Chen

**Affiliations:** ^1^College of Internet of Things Engineering, Hohai University, Changzhou, China; ^2^Changzhou City Key Lab of Orthopaedic Implants Digital Technology, Changzhou, China

## Abstract

Femur parameters are key prerequisites for scientifically designing anatomical plates. Meanwhile, individual differences in femurs present a challenge to design well-fitting anatomical plates. Therefore, to design anatomical plates more scientifically, analyses of femur parameters with statistical methods were performed in this study. The specific steps were as follows. First, taking eight anatomical femur parameters as variables, 100 femur samples were classified into three classes with factor analysis and Q-type cluster analysis. Second, based on the mean parameter values of the three classes of femurs, three sizes of average anatomical plates corresponding to the three classes of femurs were designed. Finally, based on Bayes discriminant analysis, a new femur could be assigned to the proper class. Thereafter, the average anatomical plate suitable for that new femur was selected from the three available sizes of plates. Experimental results showed that the classification of femurs was quite reasonable based on the anatomical aspects of the femurs. For instance, three sizes of condylar buttress plates were designed. Meanwhile, 20 new femurs are judged to which classes the femurs belong. Thereafter, suitable condylar buttress plates were determined and selected.

## 1. Introduction

Orthopaedic surgeons often use anatomical plates to treat bone fractures [1]. Therefore, there has been an accelerated drive to design, develop, and manufacture anatomical plates in recent years. However, significant differences in femoral sizes and shapes are manifest across gender, age, race, region, and so forth. These differences present a big challenge for the design of well-fitting anatomical plates for the mass market. During a surgical operation, the clinician has to implement trimming and reshaping repeatedly to address the poor match between the selected plate and the actual bone. Therefore, new methods are greatly needed to conveniently design anatomical plates that match bones well.

Anatomical information of the bone is the basis for the design of anatomical plates. Thus, analysis of bone parameters is very important and essential. In recent years, many scholars have carried out studies of bone parameters. Dong and Zheng [2] proposed a computational framework based on particle filtering to estimate the morphological parameters of the proximal femur. Mahaisavariya et al. [3] calculated inner and outer parameters of proximal femurs using computerized tomography (CT) images combined with the reverse engineering technique. Lv et al. [4] analysed relationships between eight morphological parameters of the proximal femur. Although they have only focused on the bone parameters level, description of statistical shape models for bones has also gained a lot of attention from many researchers. van de Giessen et al. [5] developed a quantitative, standardized description of the variations in the scaphoid and lunate by constructing a statistical shape model (SSM) of healthy bones. The SSM can provide a description of possible shape variations and the distribution of scaphoid and lunate shapes in a population. Additionally, an articulating ulna surface for prosthesis design was detected [6]. Then, this articulating surface was attached to an SSM of the ulna head, allowing the detection of articulating surfaces in ulnae that were not in the training set of the model.

The femur is the bone that is most commonly fractured. Thus, we will focus on analyses of femurs for anatomical plate design. While a customized plate is designed using an individual's own anatomy, a general plate can be naturally designed using an average femur model of a specific population. The average model can be easily achieved with advanced statistical methods [7, 8] in combination with three-dimensional (3D) medical imaging technologies [9, 10] and 3D reconstruction technology [11, 12]. If femurs in the same population can be classified into different classes, then femurs in the same class have nearly the same anatomical characteristics. Then, an average anatomical plate designed based on the average parameters of femurs in the same class is entirely reasonable. The benefit is that the designed plate can be better contoured and bent to follow the anatomy of femurs in their target population. Therefore, the amount of reshaping and trimming done during surgery can be minimized to an extent.

The main aims of this paper are twofold. First, it aims to classify femurs into different classes with advanced statistical methods. Second, it aims to design average anatomical plates with different sizes to be suitable for femurs in the different classes. Then, for a new femur, judge which class that femur would fall into based on Bayes discriminant analysis, thereby allowing a suitable anatomical plate for the new femur to be determined. To achieve these aims, statistic methods (such as factor analysis, Q-type cluster analysis, and Bayes discriminant analysis) and software (such as Mimics and Catia) were used. Experiment showed that femurs are rationally classified into three classes. The condylar buttress plate was taken as an example to illustrate the design of anatomical plates based on classified femurs.

## 2. Materials and Methods

### 2.1. Samples

**Figure 1:**
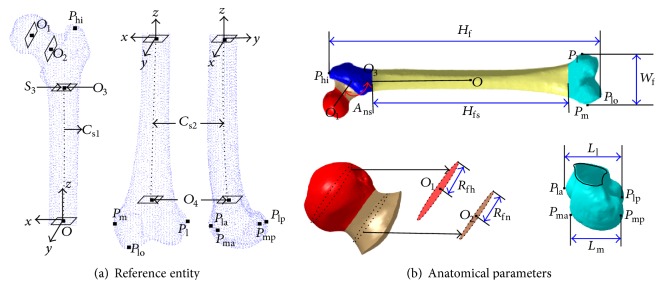
Parameters defined on reference entity.

To analyse anatomical information of femurs, anatomical parameters are necessary. These parameters include the height of the total femur (*H*_f_), the neck-shaft angle (*A*_ns_), the width of the femoral condyle (*W*_f_), the radius of the femoral head (*R*_fh_), the radius of the femoral neck (*R*_fn_), the height of the femoral shaft (*H*_fs_), and the medial anterior-posterior (*L*_m_) and lateral anterior-posterior (*L*_l_) widths. All of these parameters are based on geometrical elements previously defined in reference object [13–15]. As shown in Figure 1, the origin of coordinate *O* is located at the central point of the femoral shaft. The *x*-axis is towards the inner thigh, the *y*-axis is towards the back of thigh, and the *z*-axis is towards the proximal femur. The anatomical features points as defined in the literature are specifically described in Table 1. The eight anatomical parameters are specifically described in Table 2.

In this study, a total of 100 unrelated healthy adults, who belonged to the Chinese Han ethnic group, were voluntarily enrolled. After the subjects were informed about associated risks, a questionnaire was given to obtain the subject's age, sex, medical history, and physical activity, under the direction of a clinician. We adopted the exclusion criteria detailed elsewhere [16] to screen and recruit “healthy” subjects. To be specific, participants meeting one of the following requirements were excluded from our study: (1) women who were pregnant, breastfeeding, or planning to get pregnant; (2) individuals with a history of diseases or therapies that might potentially affect bone mass, structure, or metabolism [16]; and (3) individuals with genetic relationships, such as parent-child relationships and sibling relationships.

**Figure 2:**
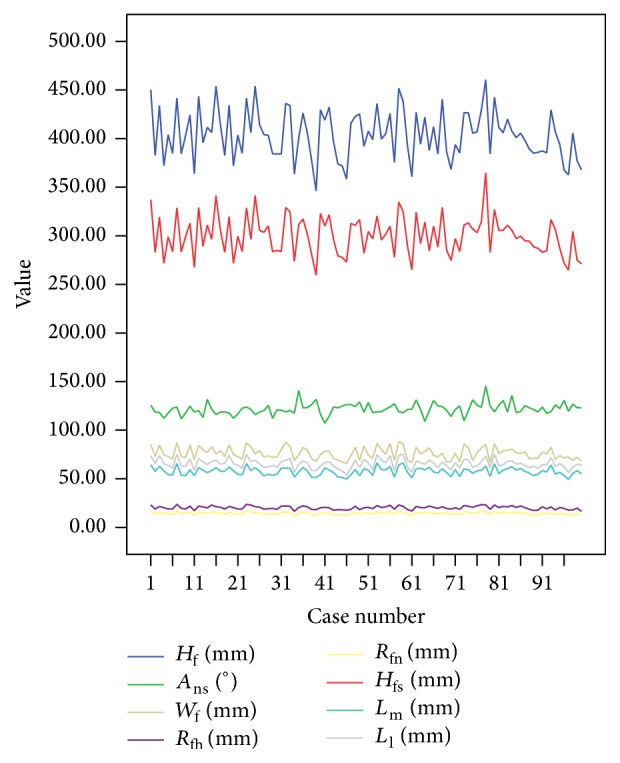
Anatomical parameters of 100 femur samples.

After the demographics and medical history were obtained, each subject's femur CT images were imported into Mimics software version 15.0 (Materialise, Belgium). Thereafter, femoral contours were segmented and a three-dimensional (3D) model was calculated based on these contours. Finally, eight anatomical parameters of each reconstructed 3D model were measured and are depicted in Figure 2.

Due to the fact that factor analysis will be used to analyse the femur parameters, Kaiser-Meyer-Olkin (KMO) measure [17, 18] of sampling adequacy and Bartlett's test of sphericity [17, 18] were used to confirm the adequacy of the factor analysis. The KMO test was used to compare the simple correlation coefficient and partial correlation coefficient between variables. The closer this value to 1 is, the stronger the correction between variables is. In Bartlett's test of sphericity, a sig. <0.05 indicates a strong correlation between variables. As shown in Table 3, a KMO statistic of 0.725 and sig. = 0 were obtained, showing strong correlations between parameters. This indicated that the femur data meet the conditions of factor analysis well.

**Figure 3:**
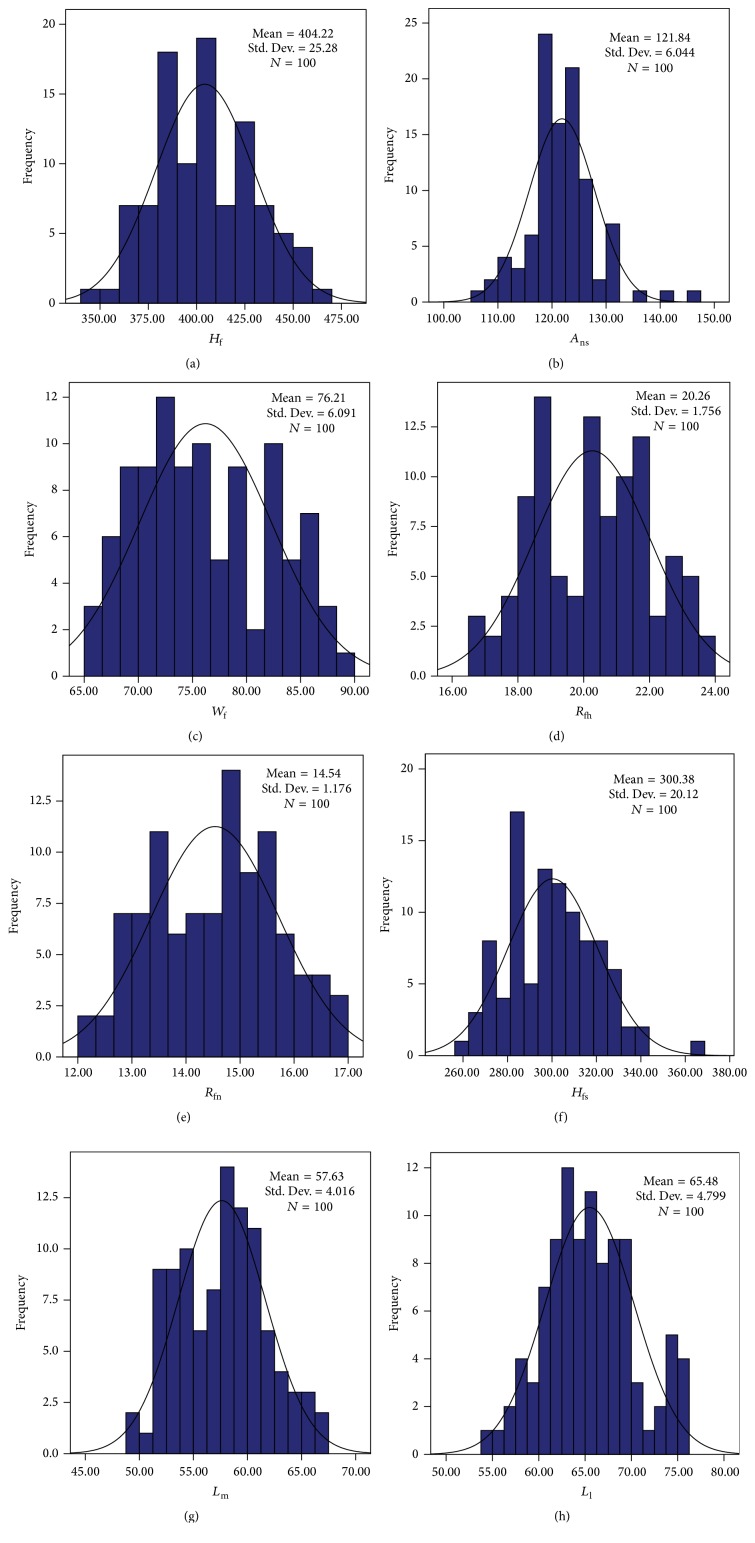
Histograms and normal curves of eight parameters.

In addition, histograms and normal curves for the variables are intuitively described in Figure 3. Additionally, the probability density function for each variable can be expressed as $p\left(x_{i}\right)=\left(1/\sqrt{2\pi }\sigma_{i}\right)\exp\left(-{\left(x-\mu_{i}\right)}^{2}/2{\sigma_{i}}^{2}\right)$, *i* = 1,2,…, 8, where *μ*_*i*_ and *σ*_*i*_ represent the mean and standard deviation of the same variable, respectively. For the values of *μ*_*i*_ and *σ*_*i*_ for each variable, please refer to Figure 3.

### 2.2. Research Method

**Figure 4:**
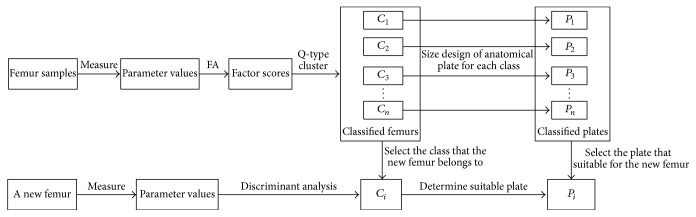
Study workflow.

The above tests showed that the sample femur data not only obeyed normal distribution but also had strong correlations. Thus, factor analysis, cluster analysis, and discriminant analysis could be conducted. As shown in Figure 4, the study workflow is as follows. First, based on the sample data, femur samples were classified into different classes using factor analysis and Q-type cluster analysis. Then, anatomical plates with different sizes were designed based on the mean parameters of each class of femurs. Thereafter, based on Bayes discriminant analysis, a new femur could be assigned to its appropriate class. Finally, the anatomical plate with a size suitable for the new femur was selected from the designed plates based on the femur's assigned class.

To express the method more concisely and clearly, the specific steps are listed as follows.

**Step 1 .**

Factors are extracted and factor scores are calculated using factor analysis [19].

*Step 1.1*. *k* (*k* < 8) independent factors are extracted to present the eight original variables along with principal component analysis (PCA) of the variables.

*Step 1.2*. To make the factors more explanatory, the factors are rotated with the varimax method.

*Step 1.3*. Factor scores for each femur sample are calculated and then saved as new variables.

**Step 2 .**

Using Q-type clustering [17], femur samples are classified into different classes based on the new variables calculated in Step 1.3.

*Step 2.1*. 100 samples are regarded as 100 classes.

*Step 2.2*. By calculating the squared Euclidean distance [17] between any two samples, the two with the nearest distances are merged into a new class with this consolidation method. At this time, there are 99 classes.

*Step 2.3*. With Ward's method [17], the distances between the new class and the other classes are calculated. Then, the two classes with the nearest distance are merged into a new class. This step is repeated until all the samples are merged into one class. The expected end result is that the sum of squares of deviations between femur samples in the same class is as small as possible and the sum of squares of deviations between classes is as large as possible.

**Step 3 .**

Based on our knowledge of anatomy, average anatomical plates are designed for each class of femurs.

*Step 3.1*. For each class of femurs, the parameters for undersurface of an average anatomical plate are designed based on average parameters of a femur of that class.

*Step 3.2*. The undersurface is thickened to be a plate.

**Step 4 .**

Based on Bayes discriminant analysis [20], a new femur is assigned to its appropriate class. From this, an anatomical plate with suitable size is determined.

*Step 4.1*. Bayes discriminant functions for each class are established based on the existing classification characteristics of the 100 femur samples.

*Step 4.2*. The parameters of the new femur are submitted to the Bayes discriminant functions. Then, the class to which the new femur belongs is determined.

*Step 4.3*. The average anatomical plate suitable for the class to which the new femur belongs is determined.

## 3. Results and Discussion

### 3.1. Classification Results

**Figure 5:**
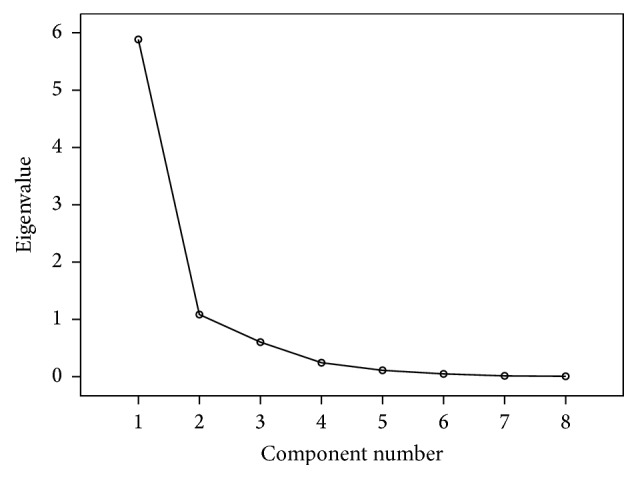
Scree plot.

**Figure 6:**
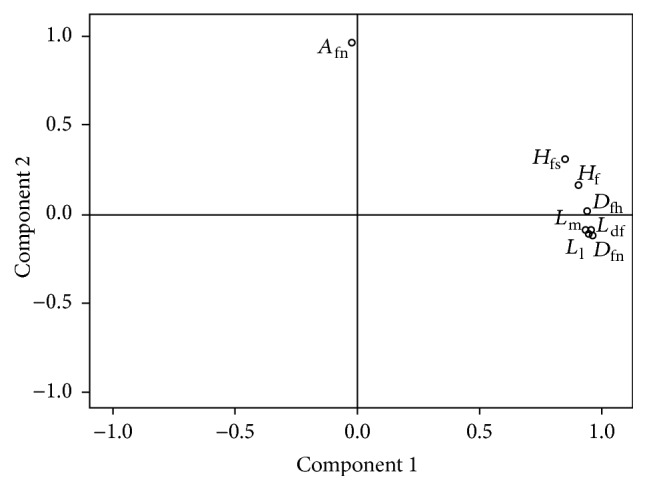
Component plot in rotated space.

During factor analysis, important factors were determined using the PCA extraction method. The effect of the extracted components on the original variable is evaluated by eigenvalues (also called variance). The values of eight eigenvalues can be clearly seen in the scree plot (see Figure 5). The changing trends of the first two eigenvalues were much larger than that of the remaining six eigenvalues. Therefore, these two components were selected for this study. Meanwhile, in order to make the explanation of the factors clearer, the factors were further rotated. From the component plot in rotated space (see Figure 6), it is intuitively seen that component 1 mainly explains *H*_f_, *W*_f_, *R*_fh_, *R*_fn_, *H*_fs_, *L*_m_, and *L*_l_, and component 2 mainly explains *A*_ns_. Based on anatomy, the first two factors can be called “size factor” and “angle factor,” respectively.

Based on the rotated component matrix (see Table 4), the mathematical model of factor analysis, that is, the correlation between the original variables and the final factors (resp., marked as *f*_1_ and *f*_2_), is expressed as

(1) Hf=0.892f1+0.161f2Ans=−0.031f1+0.96f2Wf=0.936f1−0.113f2Rfh=0.929f1+0.013f2Rfn=0.944f1−0.124f2Hfs=0.835f1+0.3f2Lm=0.93f1−0.098f2Ll=0.946f1−0.096f2.

It needs to be emphasized that the original variables are all standardized.

To further present the factors' explanatory ability for the original variables, the percent of variance was used. The higher the percent of variance, the stronger the explanatory ability the extracted factors have. According to the accumulative variance contribution shown in Table 5, the rotation sums of squared loadings of the first two principal components are 87.11%, showing that the first two components were enough to capture the vast majority of femur information.

**Figure 7:**
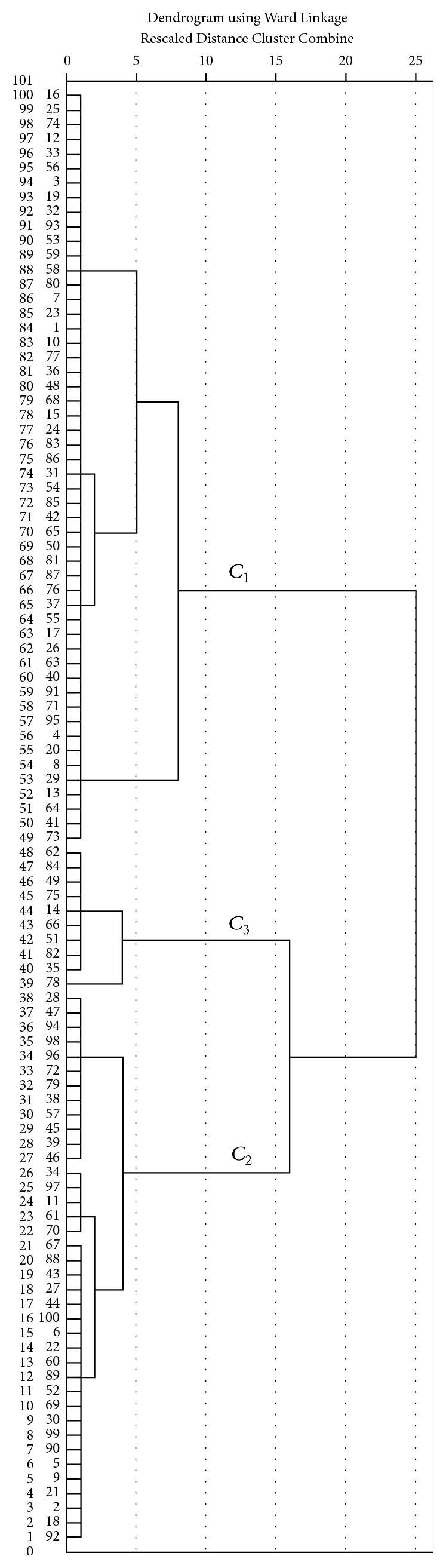
The dendrogram for hierarchical cluster.

According to the dendrogram (Figure 7), the 100 femur samples could be classified into three classes: *C*_1_, *C*_2_, and *C*_3_. The numbers in each class were 52, 38, and 10, respectively. To further understand the characteristics of each class, descriptive statistics (including mean, sample numbers, and standard deviation) of the eight variables were calculated and tabulated in Table 6. Through analysis of the classified femurs, a pattern was found describing the difference in “size factor” and “angle factor” between the different classes. For *C*_1_, *A*_fn_ was below the overall average of the 100 samples. However, the remaining seven parameters were above the sample average. For *C*_2_, *A*_fn_ was above the overall average, while the remaining seven parameters were below the sample average. For *C*_3_, all parameters were above the overall sample average. We can rationalize these findings as follows: for tall people, “size factor” is generally larger than the population average, while the “angle factor,” as a measure of polarization, is typically below the population average and only a small portion of tall people have an above average value. For short people, the “size factor” is generally below the population average, but the “angle factor” is above average.

Although significant differences between different classes can be seen in Table 6, analysis of variance (ANOVA) was used to further judge the rationality of the classification. As shown in Table 7, the sig. for each variable equals zero, showing significant differences between variables in the different classes of femurs. This shows the rationality of the classification achieved with factor analysis and Q-type clustering.

### 3.2. Design of Anatomical Plates

**Figure 8:**
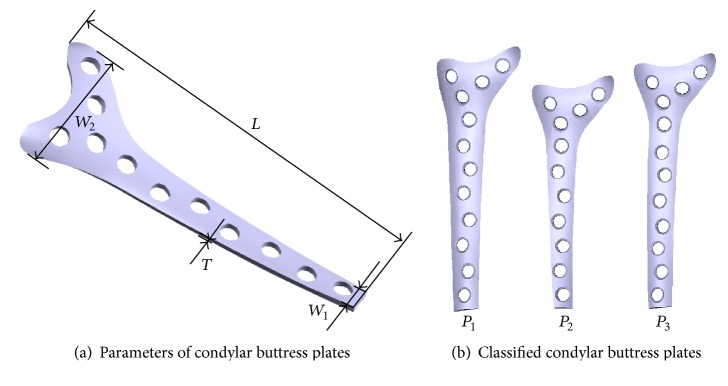
Classified condylar buttress plates.

Usually, the type of anatomical plate used for the treatment of a femoral fracture is decided according to the surgeon's clinical experience. A condylar buttress plate [21, 22], often used to treat fractures of the distal femur, was taken as an example to illustrate the design of average plates based on our different classes of femurs. As shown in Figure 8(a), the parameters used to define a condylar buttress plate include the total length (*L*), the total width of the proximal part (*W*_1_), the total width of the distal part (*W*_2_), and the thickness (*T*). Based on Catia V5R19 [23], three average condylar buttress plates (*P*_1_, *P*_2_, and *P*_3_) were designed, respectively, for our three classes of femurs (*C*_1_, *C*_2_, and *C*_3_) (see Figure 8(b)). The parameter values for each condylar buttress plate were designed based on the average parameters of the femurs in their assigned class. This was combined with surgical experience and production experience, with the final parameter values of the three condylar buttress plates shown in Figure 8(b) and detailed in Table 8.

For a new femur, to judge which class the new femur belongs to, Bayes discriminant analysis was used in this study. Based on the classification function coefficients in Table 9, Bayes discriminant functions were expressed as follows:

 For *C*_1_, *F*_1_ = −737.473 + 3.994*H*_f_ + 7.690*A*_ns_ + 0.485*W*_f_ − 37.118*R*_fh_ + 63.033*R*_fn_ − 4.225*H*_fs_ + 0.381*L*_m_ − 0.636*L*_l_;
 For *C*_2_, *F*_2_ = −712.929 + 3.882*H*_f_ + 8.171*A*_ns_ + 0.175*W*_f_ − 42.538*R*_fh_ + 66.760*R*_fn_ − 4.107*H*_fs_ + 0.352*L*_m_ − 0.535*L*_l_;
 For *C*_3_, *F*_3_ = −788.583 + 3.469*H*_f_ + 8.389*A*_ns_ + 0.265*W*_f_ − 37.980*R*_fh_ + 63.408*R*_fn_ − 3.591*H*_fs_ + 0.387*L*_m_ − 0.453*L*_l_.

Values of *F*_1_, *F*_2_, and *F*_3_ for a new femur can be calculated by substituting the femur parameters into the related formula. Among *F*_1_, *F*_2_, and *F*_3_, if *F*_1_ is the largest one, then the femur belongs to *C*_1_; if *F*_2_ is the largest one, then the femur belongs to *C*_2_; if *F*_3_ is the largest one, then the femur belongs to *C*_3_. From Table 10, we can see that the judgement accuracy of the original group of femurs was 100%, showing a high credibility for the discrimination function. Meanwhile, 20 new femurs were subjected to this classification system; of note, it is important to emphasize here that if a femur is fractured and needs repair with a plate, then the femur that would be submitted to this classification system would be the contralateral, unfractured femur.

## 4. Discussion

To design anatomical plates (such as condylar buttress plates) for femurs more scientifically, femur parameters were analysed with statistical methods in this study. Femurs were classified into three classes based on factor analysis and Q-type cluster analysis. Then, three average condylar buttress plates, one for each class of femur, were designed. For a new femur, Bayes discriminant analysis was used to judge which class the new femur fell into. A total of 20 new femurs had their class assigned and suitable plates were determined. Experiments showed that our classification of femurs was rational and provides a scientific basis for the design of anatomical plates. The contributions of the method in this paper are twofold:

1. Femur parameters were classified into three classes with factor analysis and Q-type cluster analysis. In factor analysis, “size factor” and “angle factor” were extracted with the PCA method. This simplification was appropriate according to our knowledge of human femur anatomy. Through analysis of the classified femurs, a pattern was found, a relationship between the “size factor” and “angle factor” relative to a given person's heights. For tall people, the “size factor” is generally larger than average, while the “angle factor” (a manifestation of polarity) is typically below average, with only a small portion of tall people having an above average value. For short people, the “size factor” is generally below average, while the “angle factor” is above the average.
2. Taking condylar buttress plates as an example, three average plates, one per femur class, were designed based on the average parameters of each class. With this system, for a new femur, if we want to select a well-fitting condylar buttress plate, we only need to judge which class the new femur falls into. One nice thing about this design is that the selected condylar buttress plate can be better contoured and bent to follow the anatomy of the new femur. Thus, reshaping and trimming of the selected plate during surgery can be minimized or even avoided to an extent.

The analysis of femur parameters has several benefits for research. The average model of each class of femurs can be used as the starting point for optimizing an anatomical plate. In addition, the quantitative ratio of femurs of different classes can help to optimize the quantities of different sized plates that are manufactured. Specifically, in this study, the quantitative ratio of *C*_1_, *C*_2_, and *C*_3_ was 26, 19, and 5, respectively. Thus, the initial quantitative ratio for the manufacture of *P*_1_, *P*_2_, and *P*_3_ could be set to the same value. Certainly, due to regional differences and the limited quantity of initial femur samples, the quantitative ratio should be continually adjusted as production continues.

However, there are some deficiencies in this study. The first is that the number of femur samples was relatively small. Although 100 samples were sufficient to describe the integral anatomy of femurs in a population, a larger sample size, as well as continued scientific and scholarly discourse, is still essential and necessary. The second is that due to the study being limited to a specific population group, characteristics of a new population group should be calculated starting from the beginning. Fortunately, with the development of advanced digital calculation methods, the calculation processes involved in this study are not a significant technical hurdle.

## 5. Conclusions

In summary, femur parameters were classified into three classes based on factor analysis and Q-type cluster analysis. Condylar buttress plates with three different sizes, one for each class of femur, were designed. Meanwhile, a new femur could be analysed and assigned to its appropriate class. Finally, the most suitable condylar buttress plate was selected based on the assigned class. Considering the potential value of this study, assessment and optimization of the biomechanical properties of the designed condylar buttress plates with finite element analysis still need to occur. In addition, due to space limitations, only condylar buttress plates were used to illustrate the design of anatomical plates based on our classification scheme. Thus, further experimentation needs to be more extensive, for example, analysing other types of long bones in humans to be able to scientifically design other types of anatomical plates or even intramedullary nails.

## Figures and Tables

**Table 1:** Feature points in reference entity.

Points	Description
*O*_1_	The central point of femoral head.
*O*_2_	The central point of femoral neck.
*O*_3_	The central point of the interface between femoral trochanter and femoral shaft.
*O*_4_	The central point of the interface between femoral shaft and femoral condyle.
*P*_hi_	The highest point of femoral trochanter.
*P*_m_	The pit of medial condyle of femur.
*P*_l_	The convex point of lateral condyle of femur.
*P*_lo_	The lowest point of medial condyle of femur.
*P*_ma_	The anterior medial condyle point.
*P*_mp_	The posterior medial condyle point.
*P*_la_	The anterior lateral condyle point.
*P*_lp_	The posterior lateral condyle point.

**Table 2:** Description of femur parameters.

Parameters	Description
*H*_f_	The distance between *P*_hi_ and *P*_lo_ in *z*-axis.
*A*_ns_	The angle between the line *OO*_3_ and the line *O*_1_ *O*_2_.
*W*_f_	The distance between *P*_m_ and *P*_l_.
*R*_fh_	The average value of distances from *O*_1_ to some key points on the surface of femoral head.
*R*_fn_	The average value of distances from *O*_2_ to some key points on the surface of femoral neck.
*H*_fs_	The vertical distance between *O*_3_ and *O*_4_ in *z*-axis.
*L*_m_	The distance between *P*_ma_ and *P*_mp_.
*L*_l_	The distance between *P*_la_ and *P*_lp_.

**Table 3:** KMO and Bartlett's test.

KMO and Bartlett's test	
Kaiser-Meyer-Olkin measure of sampling adequacy	0.788
*Bartlett's test of sphericity*	
Approx. chi-square	1368.567
df	28
Sig.	0.000

**Table 4:** Rotated component matrix.

	Rotated component matrix^a^	
	Component	
	1	2
*H*_f_	0.892	0.161
*A*_ns_	−0.031	0.960
*W*_f_	0.936	−0.113
*R*_fh_	0.929	0.013
*R*_fn_	0.944	−0.124
*H*_fs_	0.835	0.300
*L*_m_	0.930	−0.098
*L*_l_	0.946	−0.096

Extraction method: PCA.

Rotation method: varimax with Kaiser normalization.

^a^Rotation converged in 3 iterations.

**Table 5:** Total variance explained.

Component	Initial eigenvalues			Extraction sums of squared loadings			Rotation sums of squared loadings		
	Total	% of variance	Cumulative %	Total	% of variance	Cumulative %	Total	% of variance	Cumulative %
1	5.884	73.548	73.548	5.884	73.548	73.548	5.884	73.546	73.546
2	1.085	13.561	87.110	1.085	13.561	87.110	1.085	13.564	87.110
3	0.603	7.542	94.651						
4	0.244	3.053	97.704						
5	0.112	1.396	99.101						
6	0.049	0.615	99.715						
7	0.015	0.190	99.905						
8	0.008	0.095	100.000						

Extraction method: PCA.

**Table 6:** Descriptive statistic report for three classes of femurs.

Ward method		*H*_f_ (mm)	*A*_ns_ (°)	*W*_f_ (mm)	*R*_fh_ (mm)	*R*_fn_ (mm)	*H*_fs_ (mm)	*L*_m_ (mm)	*L*_l_ (mm)
*C*_1_	Mean	416.9869	119.2710	80.4011	21.4592	15.3946	308.3487	60.1973	68.5485
	*N*	52	52	52	52	52	52	52	52
	Std. deviation	22.00512	4.53646	4.41977	1.07996	0.72218	17.90407	2.99996	3.66439

*C*_2_	Mean	383.5337	122.3039	70.3901	18.4421	13.3384	285.3189	53.9300	61.1437
	*N*	38	38	38	38	38	38	38	38
	Std. deviation	16.04435	3.98275	2.36992	0.80654	0.57340	13.11820	2.23255	2.74853

*C*_3_	Mean	416.4560	133.4690	76.5051	20.9436	14.6423	316.1370	58.2902	66.0311
	*N*	10	10	10	10	10	10	10	10
	Std. deviation	17.68810	5.54150	5.44721	1.36577	0.75898	18.12024	3.08894	3.47538

Total	Mean	404.2216	121.8433	76.2073	20.2611	14.5380	300.3762	57.6250	65.4829
	*N*	100	100	100	100	100	100	100	100
	Std. deviation	25.28023	6.04407	6.09063	1.75628	1.17593	20.11964	4.01587	4.79921

**Table 7:** ANOVA.

	Sum of squares	df	Mean square	*F*	Sig.
*H*_f_					
Between groups	26234.001	2	13117.000	34.354	0.000
Within groups	37035.897	97	381.813		
Total	63269.898	99			

*A*_ns_					
Between groups	1703.713	2	851.856	43.198	0.000
Within groups	1912.830	97	19.720		
Total	3616.543	99			

*W*_f_					
Between groups	2201.375	2	1100.687	72.575	0.000
Within groups	1471.113	97	15.166		
Total	3672.487	99			

*R*_fh_					
Between groups	205.028	2	102.514	99.103	0.000
Within groups	100.339	97	1.034		
Total	305.366	99			

*R*_fn_					
Between groups	92.950	2	46.475	102.578	0.000
Within groups	43.948	97	0.453		
Total	136.898	99			

*H*_fs_					
Between groups	14404.542	2	7202.271	27.215	0.000
Within groups	25670.659	97	264.646		
Total	40075.201	99			

*L*_m_					
Between groups	867.309	2	433.655	57.679	0.000
Within groups	729.282	97	7.518		
Total	1596.591	99			

*L*_l_					
Between groups	1207.178	2	603.589	54.563	0.000
Within groups	1073.034	97	11.062		
Total	2280.213	99			

**Table 8:** Parameter values of three condylar buttress plates.

Classes	Means (mm)			
	*L*	*W*_1_	*W*_2_	*T*
*P*_1_	160.0	14.0	42.0	2.0
*P*_2_	130.0	10.0	38.0	1.8
*P*_3_	150.0	12.0	40.0	2.0

**Table 9:** Classification function coefficients.

	Ward method		
	*C*_1_	*C*_2_	*C*_3_
*H*_f_	3.994	3.882	3.469
*A*_ns_	7.690	8.171	8.389
*W*_f_	0.485	0.175	0.265
*R*_fh_	−37.118	−42.538	−37.980
*R*_fn_	63.033	66.760	63.408
*H*_fs_	−4.225	−4.107	−3.591
*L*_m_	0.381	0.352	0.387
*L*_l_	−0.636	−0.535	−0.453
(Constant)	−737.473	−712.929	−788.583

**Table 10:** Classification results.

Classification results^a^						
		Ward Method	Predicted group membership			Total
			*C*_1_	*C*_2_	*C*_3_	
Original	Count	1	52	0	0	52
		2	0	38	0	38
		3	0	0	10	10
		Ungrouped cases	7	11	2	20
	%	1	100.0	0.0	0.0	100.0
		2	0.0	100.0	0.0	100.0
		3	0.0	0.0	100.0	100.0
		Ungrouped cases	35.0	55.0	10.0	100.0

^a^100.0% of original grouped cases correctly classified.
